# The Effect of the Ultrasonography Measurement Method and the Conventional Measurement Method Used in Endotracheal Tube Size Selection in the Pediatric Patient Group on Postextubation Complications and Patient Recovery

**DOI:** 10.7759/cureus.46184

**Published:** 2023-09-29

**Authors:** Umit Murat Parpucu, Nilgün Şahin

**Affiliations:** 1 Department of Anesthesiology and Reanimation, Gulhane Faculty of Health Sciences, University of Health Sciences, Ankara, TUR; 2 Department of Anesthesiology and Reanimation, Ankara Etlik City Hospital, Ankara, TUR

**Keywords:** pediatric surgery, modified aldrete scoring system, subglottic diameter, pediatrics, ultrasonography, endotracheal tube

## Abstract

Introduction: In this study, we aimed to compare the cuffed intubation tube selected with the Cole formula and tracheal ultrasonography (USG) measurement method regarding postextubation complications in providing airway patency and determine its effects on patient recovery.

Materials and method: Between 01 July 2022 and 30 June 2023, American Society of Anesthesiologists (ASA) risk group I-III, 4-6-year-old patients who underwent pediatric orthodontic surgery (multiple tooth extraction) were included in the study. Data of age, gender, weight, ASA risk group, history, Cole formula, USG measurement results used in the endotracheal tube (ETT) selection (one of the two whichever application was used), fasting time, intubation success, operation time, 30th-minute modified Aldrete recovery score (MASS), and postoperative complications due to intubation (within the first postoperative hour) were analyzed retrospectively. The patients were divided into two groups according to the method used by the anesthesiologists in selecting the ETT at the beginning of the operation. The group that used Cole formula management was named I, while the group that used the USG measurement method was called II. Intubation-related complication data of the patients in the first 1 hour postoperatively and MASS values at the 30th minute were compared between the groups.

Results: In this study, 52.5% of the cases were male (n=42), 47.5% were female (n=38), the mean age was 4.84±0.84 years, and the mean body weight was 22.56±7.58 kilogram. There was no statistically significant difference between the groups regarding age, gender, body weight, ASA score, operation time, and period without oral consumption. ETT diameter measurement values according to groups were 4.73±0.46 mm in Group I and 4.41±0.61 mm in Group II. Postoperative 30th-minute MASS values were median 7 in Group I and median 8 in Group II (p<.001). MASS values were significantly higher in the Group II patient group. Intubation-related complications (postoperative cough, stridor, laryngospasm, tachypnea, wheezing, dysphonia) were observed in Group I with a rate of 40% within the first postoperative hour, while complications were marked with a rate of 17.5% in Group II (p=0.026). Complications in group II were significantly lower.

Conclusion: In the pediatric age group, especially under the age of 6, trachea measurement with USG and ETT selection is an effective, safe, and noninvasive method compared to other conventional methods. ETT size selection with USG accelerates postoperative patient recovery and reduces the risk of intubation-related complications. In addition, inflating the tube cuff under USG guidance can prevent cuff-related complications.

## Introduction

The pediatric patient group's tracheal structure is more specific [1-3]. Under eight years of age, unlike the adult larynx, the narrowest region of the larynx is the cricoid cartilage, and the cricoid has no flexibility (rima glottidis is the narrowest region in the adult) [1]. Accordingly, choosing the appropriate endotracheal tube (ETT) in pediatric patients is crucial. Although cuffless ETTs are recommended, especially in infants and young children, the use of a cuffed or uncuffed tube is still controversial [4]. While the use of a tube that is too small (thin) causes an increase in airway pressure and a decrease in ventilation, the use of a tube that is too wide may cause complications such as laryngospasm and tracheomalacia, especially after extubation, in pediatric patients [5,6]. Choosing the suitable and appropriate intubation tube is of great importance in preventing complications that may occur in the particular pediatric airway [2].

The issue of determining the ETT diameter to be used in the pediatric patient group is still controversial today. Due to anatomical, structural, and racial differences, this situation is tried to be solved by developing various formulas including height, weight, and age of the child in the literature [3,7,8]. Generally, the COLE formula (internal diameter (mm) = (age/4) +4) is used for this purpose [3]. The use of the Cole formula is a commonplace formula and may not be fully compatible between patients. The COLE formula can be measured according to age as well as height and weight and can be used to determine the ETT diameter. Apart from these, the Broselow band, designed to estimate body weight according to length, is more useful in determining ETT than the COLE formula [9]. This tape also allows determining the child's endotracheal tube size, laryngeal mask airway size, intercostal chest drain size, and intravenous cannula size [10]. Although these methods are used in daily practice, there is no definitive method accepted in the literature. The use of ultrasonography (USG) at the perioperative point of care by anesthesiologists has helped to resolve this situation [11]. With the introduction of USG into the daily practice of anesthesia, studies have reported that the ETT diameter can be determined more individually by measuring the subglottic transverse diameter of the pediatric airways [2,7,12]. In determining the ETT diameter, USG is more useful, more personalized, and more reliable as it provides visual and numerical data [12].

In our study, in order to determine the diameter of the cuffed intubation tube applied to ensure airway patency in patients in the American Society of Anesthesiologists (ASA) I-III group, aged 4-6 years, who underwent pediatric orthodontic surgery, the comparison of patients who underwent Cole formula (performing endotracheal intubation and inflating the cuff until the leak sound stops) with those who underwent USG measurement (endotracheal intubation and cuff inflation after intubation with USG in terms of laryngeal complications after extubation and to determine the effects on patient recovery was intended.

## Materials and methods

Study design

Our study was designed as a retrospective, single-center (Dr. Sami Ulus Pediatric Health and Diseases Training and Research Hospital) efficacy study. It was initiated after the approval of the survey dated 01/09/2023 and numbered E-73799008-799-223538099. All procedures performed in our study were in accordance with the ethical standards of the institutional and/or national research committee and with the 1964 Helsinki Declaration (as revised in 2013) and its later amendments or comparable ethical standards. Preoperative consent was obtained for the use of data from all patients to be operated in our clinic.

Clinical protocol

As a clinical protocol, the Cole formula method [2] (Group I) or measurement method with USG (Group II) was used in the selection of ETT suitable for all pediatric age groups. Patient selection was made randomly in routine practice. ETT calculated according to the Cole formula method was used for the patients whose procedure sequence was odd on the operation day, and ETT calculated according to the USG method was used for the patients with an even number of procedure sequences.

An intubation evaluation form was completed for each patient, including; age, gender, weight, ASA risk group, duration of fasting, diagnosis of congenital disease, presence of genetic syndrome or anomaly, presence of neck movement restriction, intubation history, tracheostomy history, oropharyngeal surgery history, Cole formula and USG method measurement result (whichever of the two applications was used), intubation success, operation time, 30th minute Modified Aldrete Scoring System (MASS) value, and postoperative complications due to intubation (within the first postoperative hour).

**Figure 1:**
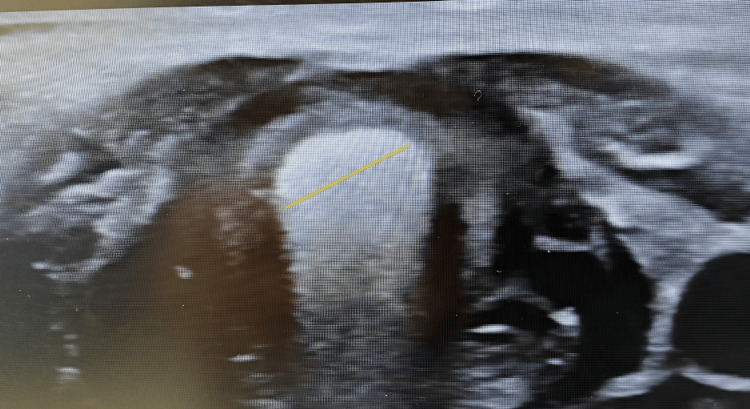
Subglottic airway measurement method with ultrasonography Yellow line: Minimal transverse diameter of subglottic airway measurement

In the Cole formula method, the ETT diameter was calculated according to age (tube internal diameter (mm) = (age/4) + 4 equation) [2]. The ultrasonographic measurement value of the transverse air column diameter at the level of the cricoid cartilage was defined as the subglottic diameter with a linear probe with a frequency of 9 MHz (Philips HDI-4000, Best, Eindhoven, The Netherlands), placed slightly in the middle of the anterior region with the neck extended (Figure 1). The ETT diameter was calculated using the measured subglottic diameter (tube internal diameter (mm) = subglottic diameter x 0.705 - 0.091) [6].

MASS is the scoring system used to determine the readiness of patients when they are sent from the recovery room to the service [13]. It is frequently used in the literature to evaluate assistive airway devices and ETT-induced postoperative recovery [14-16]. It consists of a maximum of 10 points. Evaluates the patient's activity, respiration, circulation, consciousness, and O_2_ saturation. When the MASS reaches 9 points, patients can be sent to the service from the recovery room.

Anesthesia management

All pediatric patients undergoing orthodontic operation (multiple tooth extraction) were premedicated with oral 1 mg/kg midazolam 30 minutes before the procedure. Standard monitoring with standard three-channel ECG, SpO_2_, and NIBP was performed on the patients who were taken to the operating table.

The tube size was calculated with the Cole method for Group I patients before the procedure, and the calculated tubes were prepared on the anesthesia table, together with tubes one size smaller and one size larger. During anesthesia induction, 1mcg/kg fentanyl and 2 mg/kg propofol were administered, and 0.6 mg/kg rocuronium was administered after adequate mask breathing; after 2 minutes of mask ventilation, the calculated ETT was placed after visualizing the larynx and vocal cords with the help of a video laryngoscope, after intubation, the APL valve was brought between 15 and 25 cm-H_2_O and the larynx was rested with a stethoscope in manual ventilation, and cuff inflation was performed until no leakage sound was heard.

After standard monitoring in Group II patients, sedation was provided with 1mcg/kg fentanyl on the table, and the appropriate ETT diameter was calculated by an anesthesiologist experienced in USG. The tubes, one size smaller and one larger, were prepared on the anesthesia table. After applying 2 mg/kg propofol, adequate mask breathing, 0.6 mg/kg rocuronium, and anesthesia induction, the larynx and vocal cords were visualized with the help of a video laryngoscope. After intubation was achieved with the measured ETT, the APL valve was brought to between 15 and 25 cm-H_2_O, and the endotracheal valve was inflated under manual ventilation and USG guidance.

After the surgical procedure was completed, the MASS values of the patients in the first 30 minutes after extubation and complications that might occur within the first one hour postoperatively due to intubation (postoperative cough, stridor, laryngospasm, tachypnea, wheezing, dysphonia) were recorded in the intubation evaluation form.

Patients

**Figure 2:**
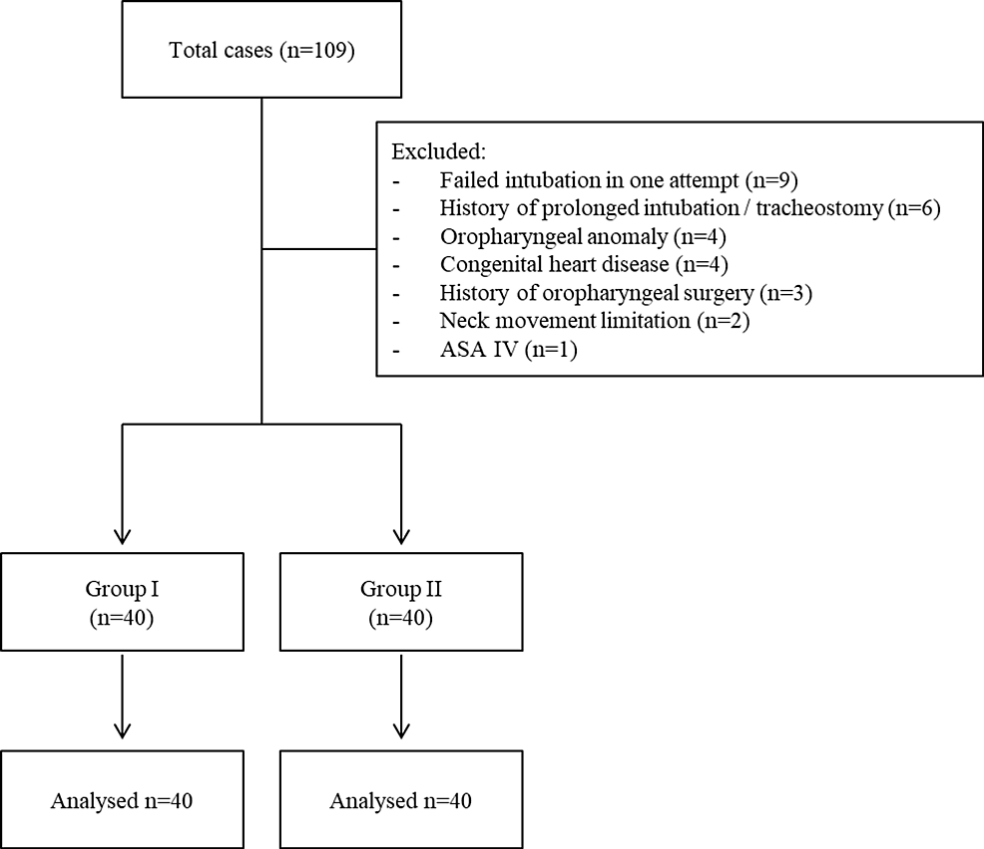
Flow charts of the patients

Our study retrospectively reviewed the data of patients who underwent multiple tooth extractions by orthodontic surgery at Dr. Sami Ulus Pediatric Health and Diseases Training and Research Hospital, between April 01, 2023 and June 30, 2023. The reason why we retrospectively analyzed the data of the patient group who only underwent multiple tooth extractions was that these were short surgical procedures that did not involve any intervention in the airway, and our article's main outcomes were not affected due to complications arising from the surgical procedure after the operation. Data of patients aged 4-6 years and in the ASA I-III risk group were included in the study. The data of patients with oropharyngeal anomaly (large tongue, cleft palate-lip, etc.) who failed intubation in one attempt (by an experienced anesthesiologist) with an ETT suitable for the calculated Cole formula tube diameter or USG measurement tube diameter the data of patients with neck motion restriction, a history of oropharyngeal surgery, a history of prolonged intubation/tracheostomy, ASA IV, and congenital heart disease were excluded from the study. The working flowchart is shown in Figure 2.

Among the patients who met the inclusion criteria, those whose ETT diameter was calculated by the Cole method were defined as Group I and those whose ETT diameter was calculated by the USG method were defined as Group II.

Outcome criteria

The primary endpoint is the comparison of postoperative complications due to intubation after the ETT, which was determined by two separate methods (Cole formula and USG measurement formula) in the 4-6 year-old ASA I-III patient group who underwent pediatric orthodontic surgery. The secondary endpoint was the MASS value differences at the postoperative 30th minute according to the ETT selection methods.

Statistical analysis

We conducted the statistical analysis for this study using IBM SPSS Statistics for Windows, Version 19 (Released 2010; IBM Corp., Armonk, New York, United States) and MedCalc version 14.12.0 (MedCalc Software, Belgium). In the statistical analysis of our study, Student's t-test was used as the parametric test, and the Mann-Whitney U test was used as the nonparametric method in the comparison of both groups. Normally distributed data are shown with mean and standard deviation (±SD), and non-normally distributed measurement data with median and 1st-3rd quartile ranges (Q1-Q3). The analysis of the count data was done with the chi-square test. p<0.05 was considered statistically significant.

## Results

Data from a total of 80 patients were included in the study (Group I, n=40; Group II, n=40); 52.5% of the cases were male (n=42), 47.5% were female (n=38), mean age was 4.84±0.84 years, and mean body weight was 22.56±7.58 kilogram. The distribution of some demographic and clinical characteristics of the patients compared to the groups is summarized in Table 1. There was no statistically significant difference between the groups regarding age, gender, body weight, ASA score, operation time, and period without oral consumption.

**Table 1:** Distribution of some demographic and clinical characteristics of the patients

Features	Group I (n=40)	Group II (n=40)	P value
Age, mean ±SD	4.68±0.82	5.0±0.84	0.087
Gender, n (%)			
-Male, n (%)	20 (50%)	22 (55%)	0.654
-Female, n (%)	20 (50%)	18 (45%)	
Body Index, kg, mean±SD	20.98±7.48	24.15±7.43	0.061
ASA score, n (%)			
- I, n (%)	8 (20%)	14 (35%)	0.139
- II, n (%)	26 (65%)	22 (55%)	
- III, n (%)	6 (15%)	4 (10%)	
Operation time, minutes	77.0±13.04	71.50±13.45	0.067
Fasting time, hours	8.10±1.91	8.78±1.60	0.092
SD: standard deviation			

ETT diameter measurement values according to groups were 4.73±0.46 mm in Group I and 4.41±0.61 mm in Group II. In contrast, the MASS values at the 30th minute after the operation were 7 in Group I and 8 in Group II; a statistically significant difference was found between the groups (p<.001, Table 2). MASS values were significantly higher in the Group II patient group.

**Table 2:** Modified Aldrete scoring system values in the postoperative recovery room at the 30th minute after the operation between the groups

MASS value*	Group I (n=40)	Group II (n=40)	P value
7, n (%)	23 (57.5%)	1 (2.5%)	< .001
8, n (%)	16 (40%)	33 (82.5%)	
9, n (%)	1 (2.5%)	6 (15%)	
MASS: modified Aldrete scoring system, *postoperative 30th minute			

When intubation-related complications observed within the first hour after the operation were examined, a statistically significant difference was found between the groups (p=0.026, Table 3), while complications were seen in 40% in Group I and 17.5% in Group II. Complications in Group II were significantly lower.

**Table 3:** Intubation-related complications in the postoperative recovery room within the first hour after the operation between the groups

Complication*	Group I (n=40)	Group II (n=40)	p-value
Yes, n (%)	16 (40%)	7 (17.5%)	0.026
No, n (%)	24 (60%)	33 (82.5%)	
*Complications: cough, stridor, laryngospasm, tachypnea, wheezing, dysphonia			

## Discussion

We performed our study retrospectively on the data of patients aged 4-6 years in the ASA I-III risk group who underwent multiple tooth extraction operations under general anesthesia and had a standardized oropharyngeal procedure. Unlike the literature studies, we used a video laryngoscope to eliminate other situations that would cause difficult intubation during intubation. In addition, we only included patients who underwent multiple tooth extraction operations under general anesthesia in our study. In this way, we aimed to minimize complications arising from the surgical procedure. We compared the Cole formula, one of the classical formulas used in intubation tube selection in surgical procedures in the pediatric patient group, with the USG method that has entered the literature in recent years.

General anesthesia is a technique that allows pediatric dentists to treat patients with severe fear and anxiety, as well as uncooperative young patients. Pediatric dentists can provide significantly better dental care by reducing patient anxiety and mobility with general anesthesia [17]. General anesthesia via endotracheal intubation can be performed for dental procedures using an oral or nasal approach. Especially in pediatric patients under six years of age, endotracheal intubation is more risky and requires experience compared to older age groups [18]. Tolerance to hypoxia is worse in young children than in adults; the funnel-like structure of the larynx requires various maneuvers and more experience during intubation [18,19]. For this reason, there are formulas for the diameters of the ETTs to be used for intubation. However, it is known that these conventional measures vary according to the race, genetic structure, and weight of the child. Subramanian et al. in Indian children reported that the best correlation in determining ETT diameter was the height of the case [20]. Hofer et al. reported that the choice of ETT size with the Broselow band is more appropriate than the age-based formula [10]. Al-Mazrou et al. showed that the age-related formula was stronger than height or weight in determining ETT diameter [21]. In a thesis study by Onuk et al. on the Turkish population, it was determined that the Cole formula determined the ETT to be greater [22]. As an alternative, appropriate diameter measurement with USG reduces tube replacement due to inappropriate intubation and minimizes post-extubation complications [12]. In our study, we compared the effect of ETT diameter determined by the Cole formula method and the USG tracheal measurement method on the development of postoperative intubation-related complications and the MASS score. We excluded patients who could not be intubated at once with the measured tube. Therefore, nine patients were excluded from the study, all of whom were measured by the Cole formula. ETT replacement was not performed in any of the patients who were measured by USG due to inappropriate tubes. We think that measurement with USG gives more reliable and accurate results in the selection of ETT in the pediatric age group.

The problematic airway becomes much more critical in anesthesia applications in the pediatric group. Since apnea tolerance is low in pediatric cases, the risk of hypoxia is high. Failure to determine the appropriate intubation tube may lead to long-term adverse outcomes such as subglottic stenosis and granuloma formation, as well as cause postoperative cough, stridor, laryngospasm, tachypnea, and wheezing [18]. Many studies have shown that USG can detect the upper airway diameter more than 80% more effectively than traditional methods, especially in the adult population [23,24]. However, no consensus meta-analysis gives an exact ratio in pediatric patients. Shibasaki et al. determined it correctly with a rate of 96% in a study they had conducted [5]. Bae et al. in their study conducted with 141 pediatric patients under eight years of age, found that the correct tube size could be selected in 60% of the cases with the USG method and in 31% of the patients with the age-based formula [6]. USG is a more helpful method than age-based formulas in determining the correct tube size; however, they stated that even USG is not a completely reliable method for determining the proper tube size in children [6]. In our study, while 40% (n=16) of postoperative complications were observed in the group detected by the Cole method, the rate of postoperative complications was 17.5% (n=7) in the group detected by the USG method. The correct tube detection rate was 82.5% with the USG method. We think that the USG method is clearly superior to the Cole method in pediatric cases.

The literature states that half of the postoperative complications occur in the first 1 hour and 75% in the first five hours, and their prognosis is worse than those in the perioperative period [4]. High ASA value, emergency surgical interventions, anesthesia applications lasting 2-4 hours, and abdominal and orthopedic surgeries have the highest risk of complications. In various studies, most complications arising from postoperative intubation are symptomatic in the first 1 hour [4-8]. However, although nausea, vomiting, and hypothermia have been examined among complications in many studies, there is no questioning of hypothermia and pain in the wake-up room in the MASS system that we use [13]. Routine analgesic medication was administered in our clinic, and every patient who came to the wake-up room was warmed with a warming blanket. In addition, the data of intubation-related complications were analyzed in our study, and postoperative patient review was evaluated with MASS. A statistically significant difference was found between the two methods used in ETT selection regarding MASS values (p=<.001). In the group selected by USG and ETT, the patients recovered significantly earlier postoperatively, and the MASS values were substantially higher. Although we think that the USG method is considerably more effective than the Cole method in choosing the appropriate tube, we recommend the choice of tube with USG for faster postoperative patient recovery.

Non-expandable cricoid cartilage is the primary cause of damage due to endotracheal intubation [21]. Therefore, subglottic measurement becomes essential. In a study by Mahran et al., they found that subglottic measurements were more accurate than supraglottic or glottic measurements in pediatric patients [25]. In our study, we measured the narrow part of the trachea by making our USG measurements in the subglottic area. For this reason, tube measurements were more objective. None of the patients whose ETT was determined by USG during intubation did not cause any strain in the subglottic area.

In the literature, USG measurements were generally made one day before while the child was awake [2,22]. In our study, we performed USG measurements under sedation on the operating table. We think that this situation increases the patient's compliance with USG and enables us to make more accurate measurements. We confirmed the tube location of the patients by reevaluating them with USG after ETT placement.

The main limitations of our study are that it was carried out in limited cases, in a single center, in a specific surgical operation group. In addition, the other disadvantage is that USG measurements were made by an experienced anesthesiologist and not evaluated by a radiologist. MASS values were assessed only at the 30th minute postoperatively and were based on a single score, and the postoperative complications of the cases were those that occurred in the first 1 hour in the postoperative care unit. The follow-up data of the cases after the first 1 hour postoperatively are unavailable. Finally, it should be noted that our study is retrospective. Further prospective studies are needed in this regard.

## Conclusions

A significant portion of postoperative complications in the pediatric patient group who will undergo surgery under general anesthesia arises from airway management. Therefore, the choice of ETT size in this age group is an important and controversial issue. Especially in children under the age of 6, tracheal measurement with USG and ETT selection is an effective, safe, and noninvasive method compared to other conventional methods. The biggest advantage of choosing the ETT size with USG over other methods is that it is patient-specific. Although proper tube selection is difficult in the pediatric age group, it reduces the rate of postextubation-related complications.

ETT size selection with USG accelerates postoperative patient recovery and reduces the risk of intubation-related complications. In addition, inflating the tube cuff under USG guidance can prevent cuff-related complications.
